# Pre-*miRNA* variants as predictors of clinical outcome in patients with squamous cell carcinomas of the nonoropharynx

**DOI:** 10.18632/oncotarget.8512

**Published:** 2016-03-31

**Authors:** Chengyuan Wang, Erich M. Sturgis, Xingming Chen, Hongliang Zheng, Qingyi Wei, Guojun Li

**Affiliations:** ^1^ Department of Otolaryngology-Head and Neck Surgery, China-Japan Friendship Hospital, Beijing, 100029, China; ^2^ Department of Head and Neck Surgery, The University of Texas MD Anderson Cancer Center, Houston, TX 77030, USA; ^3^ Department of Epidemiology, The University of Texas MD Anderson Cancer Center, Houston, TX 77030, USA; ^4^ Department of Otolaryngology-Head and Neck Surgery, Peking Union Medical College Hospital, Peking Union Medical College and Chinese Academy of Medical Sciences, Beijing 100730, China; ^5^ Department of Otolaryngology-Head and Neck Surgery, Changhai Hospital, Second Military Medical University, Shanghai 200433, China; ^6^ Duke Cancer Institute, Duke University Medical Center, Durham, NC 27710, USA

**Keywords:** pre-miRNA, polymorphisms, survival, biomarkers, head and neck cancer

## Abstract

Functional polymorphisms of *miRNAs* may affect the function and target expression of miRNAs, which can, in turn, affect the biological activity, etiology, and prognosis of cancer. We hypothesized that four common polymorphisms in pre-miRNAs (*hsa-mir*-146a rs2910164 G > C, *hsa-mir*-196a2 rs11614913 C > T, *hsa-mir*-149 rs2292832 G > T, and *hsa-mir*-499 rs3746444 A > G) are associated with survival in SCCNOP. We used univariate and multivariable Cox models to evaluate the associations between the four polymorphisms and survival. We found that *hsa-mir*-149 rs2292832 and *hsa-mir*-499 rs3746444 had statistically significant associations with survival, but *hsa-mir*-146a rs2910164 and *hsa-mir*-196a2 rs11614913 did not. Patients having the *hsa-mir*-149 CC and *hsa-mir*-499 TT wild-type genotypes had significantly better overall, disease-specific, and disease-free survival compared with those who had the corresponding variant CT/TT and CT/CC genotypes, respectively. Furthermore, these genotypes were significantly associated with reduced risk of overall death, death owing to disease, and recurrence after adjustment for important prognostic confounders, indicating that these *pre-miRNA* polymorphisms may be prognostic biomarkers for SCCNOP. Moreover, the stratified analyses based on smoking status and treatment indicated that the effects of *hsa-mir*-149 and *hsa-mir*-499 polymorphisms on survival were more pronounced in ever smokers and patients treated with chemoradiation. Our findings support that the *hsa-mir*-149 rs2292832 and *hsa-mir*-499 rs3746444 polymorphisms play a significant role in the prognosis of SCCNOP, especially in smokers and patients treated with chemoradiation. Prospective studies with larger sample sizes are needed to confirm these findings.

## INTRODUCTION

Squamous cell carcinoma of the head and neck is the sixth most common cancer worldwide [1]. One of subtypes is squamous cell carcinoma of the nonoropharynx (SCCNOP), which arises mainly from the oral cavity, hypooropharynx, and larynx. Tobacco and alcohol exposure are significant risk factors for SCCNOP, which is characterized by locally aggressive tumors, a high local recurrence rate, and a high frequency of second primary tumors. Diagnostic and therapeutic approaches for SCCNOP have improved over the past 2 decades, but the prognosis for patients with SCCNOP has not significantly improved. The survival rate differs markedly among patients with SCCNOP who had similar clinical and pathological features at diagnosis and received similar treatments. It would appear that in addition to TNM stage and smoking, inter-individual variations in genetic susceptibility may contribute to prognosis in SCCNOP. Therefore, the identification of new biomarkers that accurately predict survival in patients with this disease is needed for secondary prevention programs that include more intensive surveillance to facilitate the early detection of prognostic markers and the development of more effective treatments for patients with these biomarkers. However, such biomarkers for SCCNOP prognosis have not been established.

Recent studies have demonstrated that microRNAs (miRNAs) play key roles in a broad range of physiologic and pathologic processes and may function as tumor suppressors and/or oncogenes [2–4]. It has been shown that miRNAs influence the etiology, diagnosis, and prognosis of many cancers [4–8]. Moreover, miRNAs affect all facets of inflammation response systems such as those activated by radiotherapy-induced inflammation [9]. MiRNAs also act as key regulators in apoptotic pathways [10]. Because both inflammation response and apoptotic pathways control the mechanisms of SCCNOP response to cytotoxic therapy, genetic variation in *miRNAs* may be associated with SCCNOP outcome.

Common single-nucleotide polymorphisms (SNPs) in *miRNAs* may affect miRNA function and target expression and, in turn, biological activities and cancer etiology and prognosis. Studies have shown that the *pre-mir*-146a polymorphism may affect the miRNA expression, the *hsa-mir*-196a2 C > T and *hsa-mir*-499 A > G variant genotypes are associated with an increased risk of breast cancer, and the *pre-mir*-146a and *hsa-mir*-196a2 polymorphisms are associated with survival among lung and oropharyngeal cancer patients [11–13]. However, it is not known whether these pre*-miRNA* polymorphisms are associated with survival in patients with SCCNOP.

We found a total of 12 SNPs in the miRBase registry: 11 SNPs in the *pre-miRNA* region and 1 SNP in the mature miRNA region [14]. Of these 12 SNPs, only 5 are common SNPs (minor allele frequency > 0.05): 3 in mature *miRNA* regions (*hsa-mir*-146a rs2910164:C > G, *hsa-mir*-196-a2 rs11614913:T > C, and *hsa-mir*-499 rs3746444:A > G) and 2 in other regions of pre-*miRNAs* (*hsa-mir*-149 rs2292832:G > T and *hsa-mir*-423 rs6505162:A > C). In this study, we genotyped the 4 SNPs (rs2910164, rs11614913, rs3746444, and rs2292832) that may affect both the binding of target mRNA and the pre-*miRNA* maturation process. We did not include *hsa-mir*-423 rs6505162:C > A in this study because it did not affect hydrogen bands or predict secondary structure free energy. Therefore, in this case-series analysis of SCCNOP, the 4 SNPs in pre-*miRNAs* were selected for study. Our findings will have important prognostic implications and potentially influence individualized treatment and prevention strategies for patients with SCCNOP.

## RESULTS

### Patient characteristics

The demographic, exposure, and clinical characteristics of the 996 incident SCCNOP patients are shown in Table 1. Patient follow-up continued through July 2014. At the median follow-up time of 24.9 months, 294 deaths from any cause had been identified, of which 152 occurred as a result of SCCNOP, and 224 patients had experienced a disease relapse. Additionally, the determination of tumor HPV status indicated that the HPV DNA tumor prevalence was approximately 12.4% (chiefly HPV16) in the 432 SCCNOP patients whose tumor specimens were available for analysis; in contrast, tumor HPV positivity was up to 80.2% for oropharyngeal cancer patients in our previous study (*N* = 495) [15]. Overall, patients in the current study cohort were predominantly male (67.30%) and non-Hispanic white (93.8%).

**Table 1:** Characteristics of patients with SCCNOP (*N* = 996)

Characteristics	No. (%) of patients
Age	
≤ 57 years	410 (41.2)
> 57 years	586 (58.8)
Sex	
Male	670 (67.3)
Female	326 (32.7)
Ethnicity	
Non-Hispanic white	934 (93.8)
Other	62 (6.2)
Smoking	
Never	253 (25.4)
Ever	743 (74.6)
Alcohol use	
Never	308 (30.9)
Ever	688 (69.1)
Index cancer stage	
I or II	414 (41.6)
III or IV	582 (58.4)
Comorbidity	
None or mild	821 (82.4)
Moderate to severe	175 (17.6)
Treatment^a^	
S only	333 (33.4)
XC/XS/XCS	663 (66.6)
Death, all causes	
Yes	294 (29.5)
No	702 (70.5)
Death, owing to disease	
Yes	152 (15.3)
No	844 (84.7)
Recurrence	
Yes	224 (22.5)
No	772 (77.5)

a S, surgery; X, radiotherapy; C, chemotherapy.

### Univariate analysis of association between miRNA genotypes and survival

**Figure 1:**
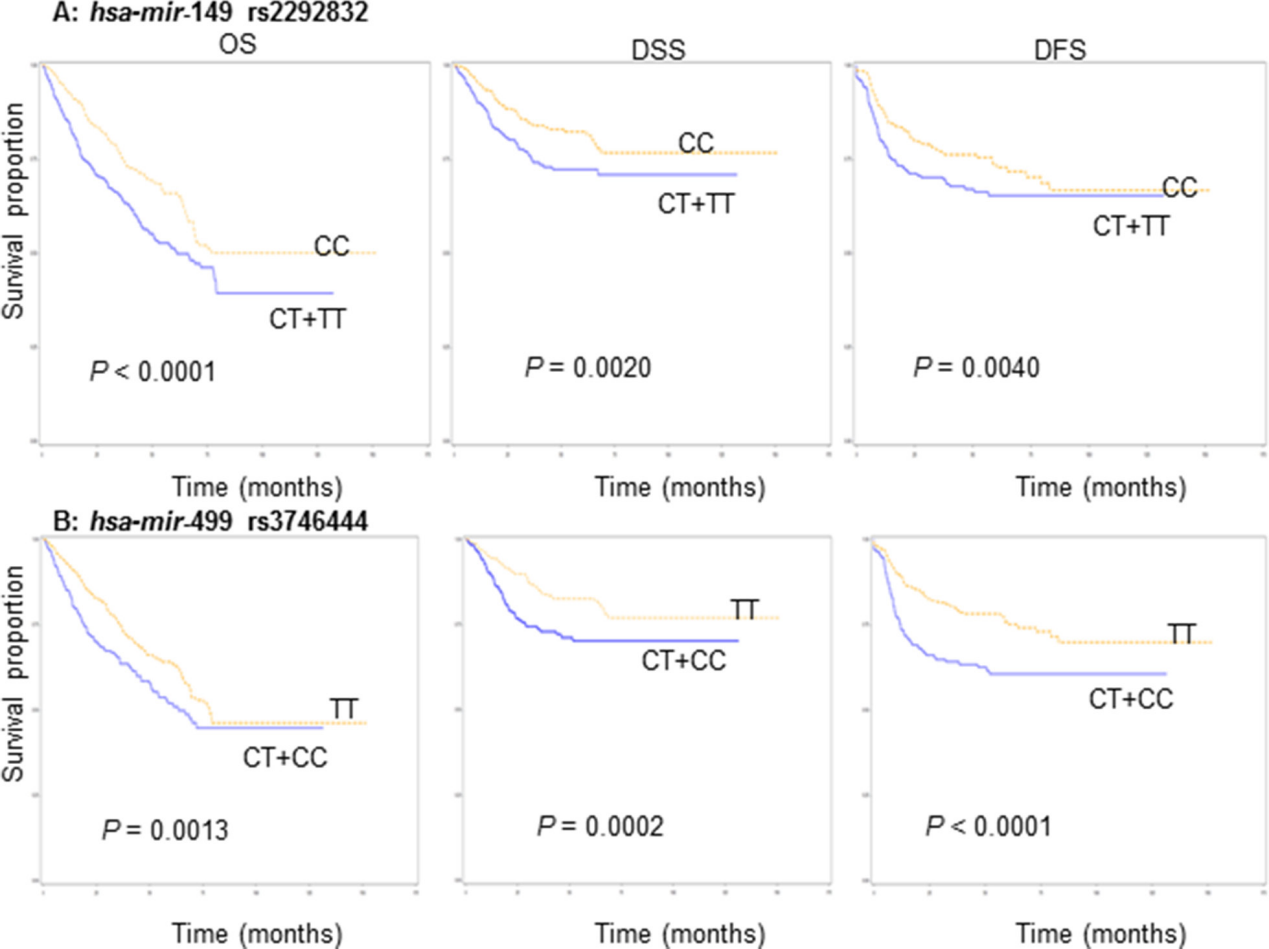
Survivals by *miRNA* genotypes in SCCNOP patients (**A**) *hsa-mir*-149 rs2292832 and (**B**) *hsa-mir*-499 rs3746444.

Survival was analyzed with respect to death from all causes (OS), death from SCCNOP (DSS), and the absence of recurrence (DFS). The results of a univariate Kaplan-Meier analysis of the association between *miRNA* genotypes and survival are shown in Figure 1. Survival analyses demonstrated statistically significant differences in OS, DSS, and DFS between carriers of *hsa-mir*-149 CC and *hsa-mir*-149 CT/TT and between *hsa-mir*-499 TT and *hsa-mir*-499 CT/CC (Figure 1). Also, SCCNOP patients carrying the *hsa-mir*-149 CC or *hsa-mir*-499 TT genotypes had better OS, DSS, and DFS than patients carrying the *hsa-mir*-149 CT/TT or *hsa-mir*-499 CT/CC genotypes, respectively. We did not, however, observe significant differences in OS, DSS, and DFS between the variant and wild-type genotypes of the other two polymorphisms (*hsa-mir*-146a and *hsa-mir*-196a2 (data not shown).

### Multivariable analysis of associations between miRNA genotypes and survival

Table 2 shows the multivariable Cox proportional hazards regression analysis of associations between *miRNA* polymorphisms and OS, DSS, and DFS in SCCNOP patients. Estimates of associations were adjusted for potential confounders, including age, sex, ethnicity, smoking and alcohol status, disease T and N stage, comorbidity, tumor subsites, and treatment. Compared with SCCNOP patients with the *hsa-mir*-149 CT/TT variant genotypes, patients with the *hsa-mir*-149 *CC* genotype had a significantly reduced risk of overall death, death from SCCNOP, and recurrence (approximately 30–40% reduction in risk). Similarly, compared with SCCNOP patients with the *hsa-mir*-499 CT/CC variant genotypes, patients with the *hsa-mir*-499 TT genotype had a significantly reduced risk of overall death, death from SCCOP, and recurrence (approximately 40–50% reduction in risk) (Table 2). For the other 2 polymorphisms (*hsa-mir*-146a and *hsa-mir*-196a2), no such significant associations were observed. Furthermore, all Kaplan-Meier survival analyses of the effects of the 4 polymorphisms were in agreement with the results from the proportional hazards regression analyses.

**Table 2:** Association of *miRNA* genotypes with OS, DSS, and DFS of SCCNOP patients (*N* = 996)

Genotypes	OS		DSS		DFS	
	Overall death/Total	HRa (95% CI)	Death, owing to disease/Total	HRa (95% CI)	Recurrence/Total	HRa (95% CI)
*hsa-mir*-146a rs2910164						
CG + CC^b^	137/453	1.0	77/450	1.0	105/453	1.0
GG	157/543	0.9 (0.7–1.2)	75/546	0.8 (0.6–1.1)	119/543	0.9 (0.7–1.2)
*hsa-mir*-196a2 rs11614913						
CT + TT^b^	153/534	1.0	80/531	1.0	114/534	1.0
CC	141/462	1.1 (0.9–1.3)	72/465	1.0 (0.8–1.4)	110/462	1.1 (0.8–1.3)
*hsa-mir*-149 rs2292832						
CT + TT^b^	159/510	1.0	84/505	1.0	121/510	1.0
CC	135/486	0.6 (0.5–0.8)	68/491	0.6 (0.4–0.8)	103/486	0.7 (0.5–0.7)
*hsa-mir*-499 rs3746444						
CT + CC^b^	131/452	1.0	77/447	1.0	120/452	1.0
TT	163/544	0.6 (0.5–0.9)	75/549	0.5 (0.4–0.8)	104/544	0.5 (0.4–0.7)

a Adjusted for age, sex, ethnicity, smoking status, alcohol use status, stage, comorbidity, and treatment.

b Reference group.

### Analysis of associations between miRNA genotypes and survival stratified by smoking status and treatment

We further evaluated the associations between *miRNA* genotypes and survival in SCCNOP patients stratified by smoking status and treatment. As shown in Table 3, in ever smokers, patients with the *hsa-mir*-149 CC and *hsa-mir*-499 TT genotypes had significantly lower risks of overall death, death from SCCNOP, and disease recurrence than patients with the corresponding variant genotypes (OS: HR = 0.7, 95% CI, 0.6–0.9, DSS: HR = 0.7, 95% CI, 0.5–0.9, and DFS: HR = 0.7, 95% CI, 0.6–0.9 for *hsa-mir*-149 and OS: HR = 0.7, 95% CI, 0.5–0.9, DSS: HR = 0.5, 95% CI, 0.4–0.8, and DFS: HR = 0.5, 95% CI, 0.4–0.9 for *hsa-mir*-499), but no significant association was found for the *hsa-mir*-146a and *hsa-mir*-196a2 polymorphisms. In never smokers, there was no significant association between any of the 4 SNPs and survival, as shown in Table 3. However, in these stratified analyses, we found that the *hsa-mir*-196a2 CC genotype was associated with an approximately 2-fold increased risk of death and disease recurrence in never smokers compared with the *hsa-mir*-196a2 CT/TT genotype, which is consistent with our previous finding that this polymorphism was significantly associated with worse outcome in HPV-associated oropharyngeal cancer patients^13^ who did not have significant tobacco exposure. It is possible that this significant association could be a biased estimate due to the small sample sizes in the stratified analysis.

**Table 3:** Association of *miRNA* genotypes with OS, DSS, and DFS of SCCNOP patients stratified by smoking status

Genotypes	OS		DSS		DFS	
	Overall death/Total	HR^a^ (95% CI)	Death, owing to disease/Total	HR^a^ (95% CI)	Recurrence/Total	HR^a^ (95% CI)
Ever smoker patients (*N* = 743)						
*hsa-mir*-146a rs2910164						
CG + CC^b^	116/346	1.0	64/343	1.0	82/346	1.0
GG	126/397	0.9 (0.7–1.1)	55/400	0.7 (0.5–1.1)	83/397	0.8 (0.6–1.1)
*hsa-mir*-196a2 rs11614913						
CT + TT^b^	136/416	1.0	69/413	1.0	94/416	1.0
CC	106/327	1.0 (0.8–1.3)	50/330	0.9 (0.7–1.3)	71/327	0.9 (0.7–1.2)
*hsa-mir*-149 rs2292832						
CT + TT^b^	124/374	1.0	65/370	1.0	86/374	1.0
CC	118/369	0.7 (0.6–0.9)	54/373	0.7 (0.5–0.9)	79/369	0.7 (0.6–0.9)
*hsa-mir*-499 rs3746444						
CT + CC^b^	112/328	1.0	61/324	1.0	85/328	1.0
TT	130/415	0.7 (0.5–0.9)	58/419	0.5 (0.4–0.8)	80/415	0.5 (0.4–0.9)
Never smoker patients (*N* = 253)						
*hsa-mir*-146a rs2910164						
CG + CC^b^	21/107	1.0	13/107	1.0	23/107	1.0
GG	31/146	1.3 (0.7–2.2)	20/146	1.4 (0.7–2.8)	36/146	1.4 (0.8–2.5)
*hsa-mir*-196a2 rs11614913						
CT + TT^b^	17/118	1.0	11/118	1.0	20/118	1.0
CC	35/135	2.0 (1.1–3.8)	22/135	2.2 (1.0–4.6)	39/135	2.0 (1.1–3.5)
*hsa-mir*-149 rs2292832						
CT + TT^b^	29/136	1.0	18/135	1.0	31/136	1.0
CC	23/117	0.9 (0.4–1.3)	15/118	0.8 (0.5–1.4)	28/117	1.0 (0.5–1.7)
*hsa-mir*-499 rs3746444						
CT + CC^b^	25/124	1.0	16/123	1.0	30/124	1.0
TT	27/129	0.9 (0.3–1.4)	17/130	0.9 (0.6–1.3)	29/129	0.8 (0.6–1.4)

a Adjusted for age, sex, ethnicity, alcohol use status, stage, comorbidity, and treatment.

b Reference group.

The results were quite similar when survival analysis was stratified by treatment, as shown in Table 4. For patients treated with radiation and/or chemotherapy with or without surgery, individuals who carried the *hsa-mir*-149 CC or *hsa-mir*-499 TT genotype had a significantly lower risk of death or disease recurrence than those with the corresponding variant genotypes (OS: HR = 0.6, 95% CI, 0.3–0.8, DSS: HR = 0.7, 95% CI, 0.5–1.0, and DFS: HR = 0.6, 95% CI, 0.3–0.9 for *hsa-mir*-149 and OS: HR = 0.6, 95% CI, 0.4–0.9, DSS: HR = 0.5, 95% CI, 0.4–0.8, and DFS: HR = 0.5, 95% CI, 0.3–0.7 for *hsa-mir*-499), while no significant association was observed for the *hsa-mir*-146a and *hsa-mir*-196a2 polymorphisms (Table 4). For patients treated with surgery only, there was no significant association between any of the 4 SNPs and prognosis. Due to either small sample sizes or clinical events in patients with SCCNOP, we did not perform a similar analysis stratified by tumor HPV16/18 status.

**Table 4:** Association of *miRNA* genotypes with OS, DSS, and DFS of SCCNOP patients stratified by treatment

Genotypes	OS		DSS		DFS	
	Overall death/Total	HR^a^ (95% CI)	Death, owing to disease/Total	HR^a^ (95% CI)	Recurrence/Total	HR^a^ (95% CI)
Patients treated with surgery only (*N* = 333)						
*hsa-mir*-146a rs2910164						
CG + CC^b^	34/147	1.0	10/147	1.0	19/147	1.0
GG	25/186	0.7 (0.4–1.2)	10/186	0.8 (0.3–2.0)	25/186	1.1 (0.6–2.1)
*hsa-mir*-196a2 rs11614913						
CT + TT^b^	29/165	1.0	10/165	1.0	20/165	1.0
CC	30/168	1.1 (0.7–1.9)	10/168	1.1 (0.5–2.8)	24/168	1.3 (0.7–2.4)
*hsa-mir*-149 rs2292832						
CT + TT^b^	31/173	1.0	11/173	1.0	23/173	1.0
CC	28/160	0.6 (0.4–1.1)	9/160	0.4 (0.1–1.1)	21/160	0.7 (0.4–1.2)
*hsa-mir*-499 rs3746444						
CT + CC	29/154	1.0	12/154	1.0	21/154	1.0
TT	30/179	0.6 (0.3–1.1)	8/179	0.5 (0.2–1.2)	23/179	0.8 (0.4–1.3)
Patients treated with chemoradiation (*N* = 663)						
*hsa-mir*-146a rs2910164						
CG + CC^b^	103/306	1.0	67/303	1.0	86/306	1.0
GG	132/357	1.0 (0.8–1.3)	65/360	0.8 (0.5–1.1)	94/357	0.9 (0.7–1.2)
*hsa-mir*-196a2 rs11614913						
CT + TT^b^	124/369	1.0	70/366	1.0	94/369	1.0
CC	111/294	1.1 (0.9–1.4)	62/297	1.0 (0.8–1.4)	86/294	1.0 (0.8–1.3)
*hsa-mir*-149 rs2292832						
CT + TT^b^	136/337	1.0	72/332	1.0	102/337	1.0
CC	99/326	0.6 (0.3–0.8)	60/331	0.7 (0.5–1.0)	78/326	0.6 (0.3–0.9)
*hsa-mir*-499 rs3746444						
CT + CC^b^	133/298	1.0	68/293	1.0	94/298	1.0
TT	102/365	0.6 (0.4–0.9)	64/370	0.5 (0.4–0.8)	86/365	0.5 (0.3–0.7)

a Adjusted for age, sex, ethnicity, smoking status, alcohol use status, stage, and comorbidity.

b Reference group.

## DISCUSSION

Our results demonstrate the association between certain pre*-miRNA* polymorphisms and survival among patients with SCCNOP. We found that patients with the *hsa-mir*-149 CC and *hsa-mir*-499 TT genotypes had a significantly lower risk of death and disease recurrence than did patients with the corresponding variant genotypes. Furthermore, we found that these significant associations were restricted to the patients who were ever smokers and the patients treated with radiation and/or chemotherapy with or without surgery. These results suggest that the *hsa-mir*-149 and *hsa-mir*-499 polymorphisms predict prognosis in patients with SCCNOP, particularly in some subgroups, which may help physicians develop individualized treatment plans that improve survival and quality of life for patients with this disease.

If the associations between these polymorphisms and SCCNOP death and disease recurrence are confirmed, clinicians could use these functional polymorphisms as biomarkers to identify an important subgroup of patients who are at high risk of death or recurrence. It is likely that future targeted therapies will be designed (in part) to counteract the effects of significant SNPs as well as individualized within the SCCNOP subgroup. Furthermore, it may be possible to intensify treatment for patients in whom the adverse genotypes are identified before treatment begins, add adjuvant therapy for those with such adverse genotypes identified immediately after treatment, and intensify the workup for treatable recurrent disease or in those who are found to have a high risk of death and recurrence in follow-up. However, these potential therapeutic options would each have to be tested in clinical trials. While local or distant recurrent SCCNOP is almost always ultimately fatal, it is hoped that the earlier recurrent disease is detected the greater the chances of successfully treating such disease. Ultimately, the data from studies such as this one will improve prognostication, facilitate more selective use of systemic therapy, and hopefully, improve outcomes.

It has been demonstrated that some *miRNAs* are upregulated in head and neck cancer cell lines and tumors, and high expression levels of these genes may be associated with cancer progression and poor outcomes [16, 17]. However, other *miRNAs* appear to be down-regulated, and their reduced expression may contribute to the development and/or progression of such cancers by coordinating a loss of sensitivity to ionizing radiation [18]. Therefore, *miRNA* expression patterns could be powerful biomarkers for the diagnosis and prognosis of head and neck cancers [17]. Genetic polymorphisms of *miRNAs* are one of the most important genetic alterations and are capable of causing reductions or increases in *miRNA* expression levels, and in turn, individual variations in the regulation of inflammatory and apoptotic responses [19].

Studies have suggested that *miRNAs* are critical in mediating inflammatory response *in vitro* and *in vivo* [20]; and they have both anti-inflammatory and pro-inflammatory actions through the regulation of inflammatory signaling pathways, targeting key elements in the pathways for the regulation of inflammatory responses to cytotoxic therapy, including chemoradiotherapy [20]. Functional analyses also have indicated that *miRNAs* are involved in the regulation of inflammatory responses and cytokine signaling [21]. On the other hand, *miRNAs* influence apoptosis through their regulation of both intrinsic and extrinsic apoptotic pathways and oncogenic and tumor suppressor networks [22]. Therefore, genetic alterations of *miRNAs* may lead to evasion of apoptosis, a mechanism that possibly leads to tumorigenesis by influencing apoptosis and cell-survival pathways through resistance to radiation. Evasion of apoptosis also may dictate the evolution of neoplastic cells. Given the roles of *miRNAs* as regulators of apoptosis, it is likely that *miRNAs* modulate sensitivity/resistance to radiation therapy in SCCNOP patients.

Several studies have investigated the associations between the 4 *miRNA* polymorphisms in this study and cancer risk and prognosis [11, 12, 23–26], but none have investigated whether these 4 polymorphisms are associated with survival in SCCNOP patients. An association between *miRNA* polymorphisms and cancer risk and prognosis is well supported by several lines of evidence. First, our previous study suggested that these 4 *pre-miRNA* polymorphisms may have a joint effect on the risk of SCCHN [27]. The SNPs located within either the pre*-miRNAs* or the *miRNA* binding sites are in theory most likely to affect the expression of the *miRNA* targets contributing to cancer susceptibility and prognosis [23, 24]. Studies have evaluated the association between these same 4 SNPs and survival in cancer patients and found that *hsa-mir*-196a2 rs11614913 CC was significantly associated with decreased survival in non-small cell lung cancer and squamous cell carcinoma of the oropharynx (SCCOP) [11, 13]. Our previous study and others also showed that both *hsa-mir*-146a rs2910164 and *hsa-mir*-196a2 rs11614913 were significantly associated with survival in SCCOP patients [13]. However, in the current study, such an association was not found in SCCNOP for these 2 SNPs, and we found instead that the *hsa-mir*-149 and *hsa-mir*-499 polymorphisms were significantly associated with survival in SCCNOP. The mechanisms by which different pre-*miRNAs* and their target mRNAs regulate progression in different types of cancer could be different, and the subject warrants further study. For example, SCCOP is mainly driven by HPV infection, whereas SCCNOP is caused by smoking [13]. Although we do not know how these pre-*miRNA* variants influence the survival of SCCNOP, it is biologically plausible that these variants are either functional or in linkage disequilibrium with other functional variants of *miRNAs* or alleles at other nearby susceptibility loci. Such functional variants could increase or reduce *miRNA* expression levels and thus affect the regulation of inflammation and apoptotic responses. The altered regulation in these pathways might enable many cancer cells to escape or counterattack the inflammatory and apoptotic responses, leading to individual variations in inflammatory/apoptotic responses to chemoradiotherapy.

The significant association was found in ever smokers but not in never smokers. This finding can be explained by the direct effect of tobacco smoking on both pro-inflammatory and immunosuppressive responses [28–30], which will influence the host response to treatment and thus affect the risk of death and disease recurrence. Smoking also has an indirect effect on inflammatory responses [31]. It has been reported that smoking can affect the expression and secretion of pro-inflammatory cytokines. Furthermore, the significant association was more pronounced in SCCNOP patients treated with chemotherapy and/or radiotherapy. This finding is biologically plausible. Radiotherapy and chemotherapy induce DNA damage in cancer cells. Patients with SCCNOP may be more likely to have somatic genetic changes, and these putatively functional variants of pre*-miRNA* might allow many tumor cells to escape from the inflammatory system and apoptotic responses and lead to different sensitivity to chemoradiotherapy, subsequently affecting the risk of death and disease recurrence. However, all these hypotheses need to be tested in future studies.

The current study has the following limitations. First, because most of the patients in this study were non-Hispanic whites, our results may not be generalizable to other ethnic groups. Second, all subjects prospectively completed a standardized epidemiologic questionnaire at enrollment that included demographic, exposure, and clinical data; however, clinical outcomes were collected retrospectively, without a strictly defined screening or follow-up regimen. Thus, future prospective studies with larger sample sizes are needed. Third, because of the hospital-based and retrospective nature of the parent study, there may have been a selection bias and confounding bias for the study patients. Finally, because of the small sample size and the limited number of outcome events in some strata, our statistically significant results might have occurred by chance. Larger studies are warranted to confirm our findings.

## MATERIALS AND METHODS

### Study patients

A total of 1197 patients with newly diagnosed, previously untreated, histopathologically confirmed index SCCNOP were consecutively enrolled in this study from October 1999 through May 2012 at our institution as part of an ongoing molecular epidemiologic study, which has been described previously [32]. All subjects were recruited regardless of age, sex, ethnicity, or clinical stage, except that patients with distant metastases at presentation were excluded. Before enrollment, all participants signed an informed consent form that was approved by the Institutional Review Board of The University of Texas MD Anderson Cancer Center. Approximately 95% of contacted patients consented to enrollment in the study. At the patients' first presentation to our institution, we collected information related to demographics, epidemiologic risk factors, and clinical characteristics, as well as blood samples for genotyping. Because of insufficient data on follow-up and/or treatment or unavailable/insufficient blood samples for genotyping, 201 enrolled participants were ultimately excluded. Therefore, our final analysis included 996 patients with previously untreated incident SCCNOP.

We followed and monitored the patients throughout their treatment and post-treatment courses through regularly scheduled clinical and radiographic examinations. Patients were considered disease free if there was no disease documented on the date of the last visit with the head and neck surgeon, head and neck radiation oncologist, or head and neck medical oncologist. There were no universal standards for imaging. Typically, patients had either routine serial imaging or follow-up imaging on the basis of symptoms or findings from physical examination. Clinical data, including stage of the index tumor at presentation, site of the index tumor, and treatment, were obtained at initial presentation and through follow-up examinations. Index cancer stage was dichotomized into early-stage (clinical stage I and II) and late-stage (clinical stage III and IV) disease. We divided treatment into 4 categories: surgery only, surgery plus radiotherapy and/or chemotherapy, radiotherapy only, and radiotherapy plus chemotherapy. We subsequently dichotomized treatment into treatment with DNA-damaging agents (radiation and/or chemotherapy) and treatment without such agents (surgery only). Medical comorbidities were classified according to a modification of the Kaplan-Feinstein comorbidity index (Adult Comorbidity Evaluation 27), which categorizes related comorbidities as none to mild, moderate, or severe. The ACE-27 grades specific diseases and conditions as 1 of 3 levels of comorbidity: grade 1 (mild), grade 2 (moderate), or grade 3 (severe), according to the severity of individual organ decompensation and the prognostic effect. Once the patient's individual diseases or comorbid conditions have been classified, an overall comorbidity score (none, mild, moderate, or severe) is assigned on the basis of the highest ranked single ailment. In cases in which 2 or more moderate ailments occur in different organ systems or disease groups, the overall comorbidity score is designated as severe. At presentation, all patients provided epidemiologic data, including alcohol drinking and smoking status.

### *miRNA* genotyping

The blood samples were used to extract genomic DNA for *miRNA* genotyping. The 4 polymorphisms of *hsa-mir*-146a (G > C, rs2910164), *hsa-mir*-196a2 (C > T, rs11614913), *hsa-mir*-499 (A > G, rs3746444), and *hsa-mir*-149 (G > T, rs2292832) were genotyped as described previously [11]. Approximately 10% of the samples were rerun, with 100% concordance.

### Tumor HPV detection

Paraffin-embedded tissue biopsies or specimens from study patients were used to extract DNA for tumor HPV16/18 detection using the specific polymerase chain reaction and *in situ* hybridization methods described previously [15]. For quality control, a subset of samples were re-assayed for tumor HPV16/18 status. The results of the re-run samples were 100% concordant with the original results.

### Statistical analysis

Statistical Analysis System software (Version 9.2; SAS Institute, Cary, NC) was used for all of the statistical analyses. The primary endpoints of the study were overall deaths, deaths due to disease, and recurrence. We investigated differences in disease-free survival (DFS), disease-specific survival (DSS), and overall survival (OS) among SCCNOP patients. Time to recurrence was computed from the date of the end of treatment to the date of last follow-up or clinical detection of recurrent cancer (local, regional, or distant). Participants who were recurrence free or lost to follow-up were censored. Overall survival was defined as the time from first appointment to death from any cause or date of last follow-up. Disease-specific survival was defined as the time from first appointment to death from disease or date of last follow-up. For both overall and disease-specific survival calculations, participants who were alive at the end of the study period or lost to follow-up were censored.

In the univariate analysis, we evaluated epidemiological variables assessed at the time of diagnosis, such as age in years, ethnicity, sex, smoking and alcohol status, and clinical characteristics, such as index tumor site, index tumor stage, treatment, and tumor subsite. Although the univariate prognostic analysis was not statistically significant for several variables, including age, sex, ethnicity, alcohol, comorbidity, and tumor subsite, these variables were retained in the main-effects and final multivariable model owing to epidemiological and clinical considerations in building the model. We also used the Kaplan–Meier method to compare survival between patients with different genotypes and calculated the log-rank statistic to test the hypothesis that there was a difference in survival between these groups. Then we investigated how genotypes modulated survival and whether the genotypes were statistically associated with survival among SCCNOP patients by fitting a Cox proportional hazards model that included age, sex, ethnicity, smoking history, alcohol consumption, disease T and N stage, comorbidity, tumor subsites, and treatment as covariates. For all analyses, statistical significance was set at *p* < 0.05, and all tests were two-sided.

## Abbreviations

miRNA: microRNA
SCCHN: squamous cell carcinoma of the head and neck;HR hazard ratio
CI: confidence interval
PCR: polymerase chain reaction
SNPs: single nucleotide polymorphisms
HPV: human papillomavirus
SCCNOP: squamous cell carcinomas of the nonoropharynx.
